# Delayed Initiation of ECMO Is Associated With Poor Outcomes in Patients With Severe COVID-19: A Multicenter Retrospective Cohort Study

**DOI:** 10.3389/fmed.2021.716086

**Published:** 2021-09-16

**Authors:** Xuyan Li, Ming Hu, Ruiqiang Zheng, Yishan Wang, Hanyujie Kang, Li Jiang, Ming Zhong, Ling Sang, Xia Zheng, Chun Pan, Wei Zhang, Haibo Qiu, Bin Du, Zhaohui Tong

**Affiliations:** ^1^Department of Respiratory and Critical Care Medicine, Beijing Institute of Respiratory Medicine, Beijing Chao-yang Hospital, Capital Medical University, Beijing, China; ^2^Department of Critical Care Medicine, Wuhan Pulmonary Hospital, Wuhan, China; ^3^Department of Critical Care Medicine, Northern Jiangsu People's Hospital, Yangzhou, China; ^4^Department of Critical Care Medicine, Xuanwu Hospital, Capital Medical University, Beijing, China; ^5^Department of Critical Care Medicine, Zhongshan Hospital, Fudan University, Shanghai, China; ^6^Department of Critical Care Medicine, Guangzhou Institute of Respiratory Health, The First Affiliated Hospital of Guangzhou Medical University, Guangzhou, China; ^7^Department of Critical Care Medicine, The First Affiliated Hospital of Zhejiang University, Hangzhou, China; ^8^Department of Critical Care Medicine, Zhongda Hospital, School of Medicine, Southeast University, Nanjing, China; ^9^Emergency Department, The 900th Hospital of Joint Service Corps of Chinese People's Liberation Army (PLA), Fuzhou, China; ^10^Medical Intensive Care Unit (ICU), Peking Union Medical College Hospital, Peking Union Medical College & Chinese Academy of Medical Sciences, Beijing, China

**Keywords:** coronavirus disease 2019, respiratory failure, acute respiratory distress syndrome, extracorporeal membrane oxygenation, outcome

## Abstract

**Background:** Extracorporeal membrane oxygenation (ECMO) is a rapidly evolving therapy for acute lung and/or heart failure. However, the information on the application of ECMO in severe coronavirus disease 2019 (COVID-19) is limited, such as the initiation time. Especially in the period and regions of ECMO instrument shortage, not all the listed patients could be treated with ECMO in time. This study aimed to investigate and clarify the timing of ECMO initiation related to the outcomes of severe patients with COVID-19. The results show that ECMO should be initiated within 24 h after the criteria are met.

**Methods:** In this retrospective, multicenter cohort study, we enrolled all ECMO patients with confirmed COVID-19 at the three hospitals between December 29, 2019 and April 5, 2020. Data on the demographics, clinical presentation, laboratory profile, clinical course, treatments, complications, and outcomes were collected. The primary outcomes were successful ECMO weaning rate and 60-day mortality after ECMO. Successful weaning from ECMO means that the condition of patients improved with adequate oxygenation and gas exchange, as shown by the vital signs, blood gases, and chest X-ray, and the patient was weaned from ECMO for at least 48 h.

**Results:** A total of 31 patients were included in the analysis. The 60-day mortality rate after ECMO was 71%, and the ECMO weaning rate was 26%. Patients were divided into a delayed ECMO group [3 (interquartile range (IQR), 2–5) days] and an early ECMO group [0.5 (IQR, 0–1) days] based on the time between meeting the ECMO criteria and ECMO initiation. In this study, 14 and 17 patients were included in the early and delayed treatment groups, respectively. Early initiation of ECMO was associated with decreased 60-day mortality after ECMO (50 vs. 88%, *P* = 0.044) and an increased ECMO weaning rate (50 vs. 6%, *P* = 0.011).

**Conclusions:** In ECMO-supported patients with COVID-19, delayed initiation of ECMO is a risk factor associated with a poorer outcome.

**Trial Registration:** Clinical trial submission: March 19, 2020. Registry name: A medical records-based study for the clinical application of extracorporeal membrane oxygenation in the treatment of severe respiratory failure patients with novel coronavirus pneumonia (COVID-19). Chinese Clinical Trial Registry: https://www.chictr.org.cn/showproj.aspx?proj=51267,identifier:~ChiCTR2000030947.

## Introduction

Severe acute respiratory syndrome coronavirus (SARS-CoV) and Middle East respiratory syndrome coronavirus (MERS-CoV) were responsible for the first two pandemics at the beginning of the Twenty-first century. The ongoing SARS-CoV-2 pandemic was the third coronavirus pandemic of this century (1). Coronaviruses are enveloped ribonucleic acid (RNA) viruses that represent relevant pathogens in the respiratory tract and lung infections (2, 3). Coronavirus disease 2019 (COVID-19) caused by SARS-CoV-2 was identified in Wuhan, China, in December 2019 (4), and it has become a global health concern. The number of cases of COVID-19 is increasing substantially worldwide.

However, the mortality rate of critically ill patients with COVID-19 was 61.5%, and patients who developed acute respiratory distress syndrome (ARDS) have been at high risk of dying from refractory hypoxemia, even with timely and standardized invasive mechanical ventilation (IMV) (5). The mortality rate has even reached 97% in patients receiving invasive ventilation (6). As the ultimate means of respiratory support, extracorporeal membrane oxygenation (ECMO) can maintain oxygenation and effectively implement a “lung protective ventilation strategy” (7). Previous studies have shown that ECMO could reduce the mortality of patients with severe ARDS in the influenza A (H1N1) outbreak of 2009 (8), avian influenza A (H7N9) viral pneumonia (9), and MERS (10). Therefore, we believe that ECMO could also be effective for other types of severe viral pneumonia (11). A recent descriptive study that compared the outcomes of patients on ECMO with mechanical ventilation to patients without ECMO but with mechanical ventilation also indicated that ECMO might be an effective salvage treatment for severe patients infected with COVID-19 (12).

Studies have shown that the mortality of severe COVID-19 patients treated with ECMO was relatively high, ranging from 57.1 to 100%, although the sample sizes of these studies were all relatively small (5, 12, 13). A large sample study in patients suffering from COVID-19 who were treated with ECMO found a 90-day mortality rate of 39% (14). Recently, several multicenter studies found that the mortality of patients with COVID-19 managed with ECMO ranged from 33.2 to 54.3% (15–17). The effect of ECMO on the outcome of patients with severe COVID-19 remains uncertain. The feasibility of launching randomized clinical trials to verify the efficacy of ECMO in this population is affected by many difficulties in patient recruitment, study design, and ethical concerns. Therefore, an observational research is a reasonable alternative. In this study, we expect to compare the effect of early and delayed initiation of ECMO on the outcomes of patients with severe ARDS related to COVID-19.

## Methods

### Study Design

This is a retrospective, multicenter cohort study. All the adult severe patients with COVID-19 treated with ECMO in Jinyintan Hospital, Wuhan Pulmonary Hospital, and Wuhan Asian General Hospital between December 29, 2019 and April 5, 2020 at Wuhan, China, were enrolled in this study. According to the time between meeting the ECMO criteria and the initiation of ECMO, the included patients were divided into two groups, namely, the “early ECMO group” and “delayed ECMO group.” The former refers to the group of patients who began ECMO within 24 h of meeting the ECMO to Rescue Lung Injury in Severe ARDS (EOLIA) trial criteria (7), as indicated by one of the following three criteria: Partial pressure of arterial oxygen [PaO_2_]/fraction of inspired oxygen [FiO_2_] ratio of <50 mmHg for more than 3 h; PaO_2_/FiO_2_ ratio of <80 mmHg for more than 6 h; or an arterial blood pH of <7.25 with a partial pressure of arterial carbon dioxide of at least 60 mmHg for more than 6 h. The “delayed ECMO group” refers to the group of patients that did not begin ECMO until more than 24 h after meeting one of the first indications for ECMO initiation. The effect of the timing of ECMO initiation on the outcomes of patients was investigated by comparing the groups.

The study was approved by the Jinyintan Hospital ethics board (KY-2020-10.02). Informed consent was waived since we collected and analyzed all data from the patients according to the policy for public health outbreak investigation of emerging infectious diseases issued by the National Health Commission of the People's Republic of China.

### Study Population

The inclusion criteria were the adult patients with ARDS (≥18 years old) who had laboratory-confirmed COVID-19 and were given veno-venous ECMO (VV-ECMO) support because of refractory hypoxemia (PaO_2_/FiO_2_ ratio <50 mmHg for 3 h and PaO_2_/FiO_2_ ratio <80 mmHg for 6 h). The diagnosis of ARDS was made according to the Berlin definition (18). The confirmed diagnosis of COVID-19 was established according to the definition established by the WHO interim guidance with positive SARS-CoV-2 nucleic acid test results in throat swab specimens (19).

Due to the limitations of the resources and professional staffing for ECMO during the early stage of the pandemic, only three representative hospitals were included in this study. The exclusion criteria were an age of more than 75 years; receipt of mechanical ventilation >7 days; and multiple organ failure (such as severe liver failure, massive upper gastrointestinal hemorrhage, and disseminated intravascular coagulation). Given the poorer outcome with age, the balance between resource availability and the potential to improve outcomes should be considered. Because the outcome worsens with time on invasive mechanical ventilation (IMV), patients on mechanical ventilation >7 days should also be excluded (note: this is a general guideline that may not apply to specific patients with COVID-19 depending on the local circumstances). In addition, the use of ECMO in patients with a combination of advanced age, multiple comorbidities, or multiple organ failure should be rare, and no such patients were included in the study.

### Data Collection and Outcomes

In this study, we retrospectively collected data on the demographics, clinical presentation, laboratory results, time from onset to ICU admission, duration of high-flow nasal cannula (HFNC) therapy before ECMO, duration of non-invasive ventilation (NIV) before ECMO, duration of IMV before ECMO, implementation of rescue ventilation strategies (such as prone-position ventilation) before ECMO, disease severity score, total duration of ECMO and IMV, and treatments and complications during ECMO. The primary outcomes were successful ECMO weaning rate and 60-day mortality after ECMO. Successful weaning refers to a group of patients whose condition improved with adequate oxygenation and gas exchange, as shown based on the vital signs, blood gases, and chest x-ray results, and who were weaned from ECMO for at least 48 h. All included patients were followed up until intensive care unit (ICU) discharge or death, or up to June 15, 2020. Two researchers independently reviewed the case report form to double check and input the collected data.

### Statistical Analysis

Descriptive data were presented as frequencies (percentages) for discrete variables and as the means (SDs) or medians [interquartile ranges (IQRs)] for continuous variables. Comparisons were determined by Student's *t*-test and the Mann–Whitney test for continuous variables and by the use of Fisher's exact test for categorical variables. Repeated measured data were compared by repeated measured ANOVA. The Kaplan–Meier curves between groups were compared by the log-rank test. The statistical significance level was set at 0.05 (two-tailed). All the analyses were conducted with SPSS version 23.0 (SPSS Inc., Chicago, IL, USA) and GraphPad Prism 8 (San Diego, CA, USA).

## Results

### Demographics and Clinical Course of Patients Treated With ECMO

**Figure 1:**
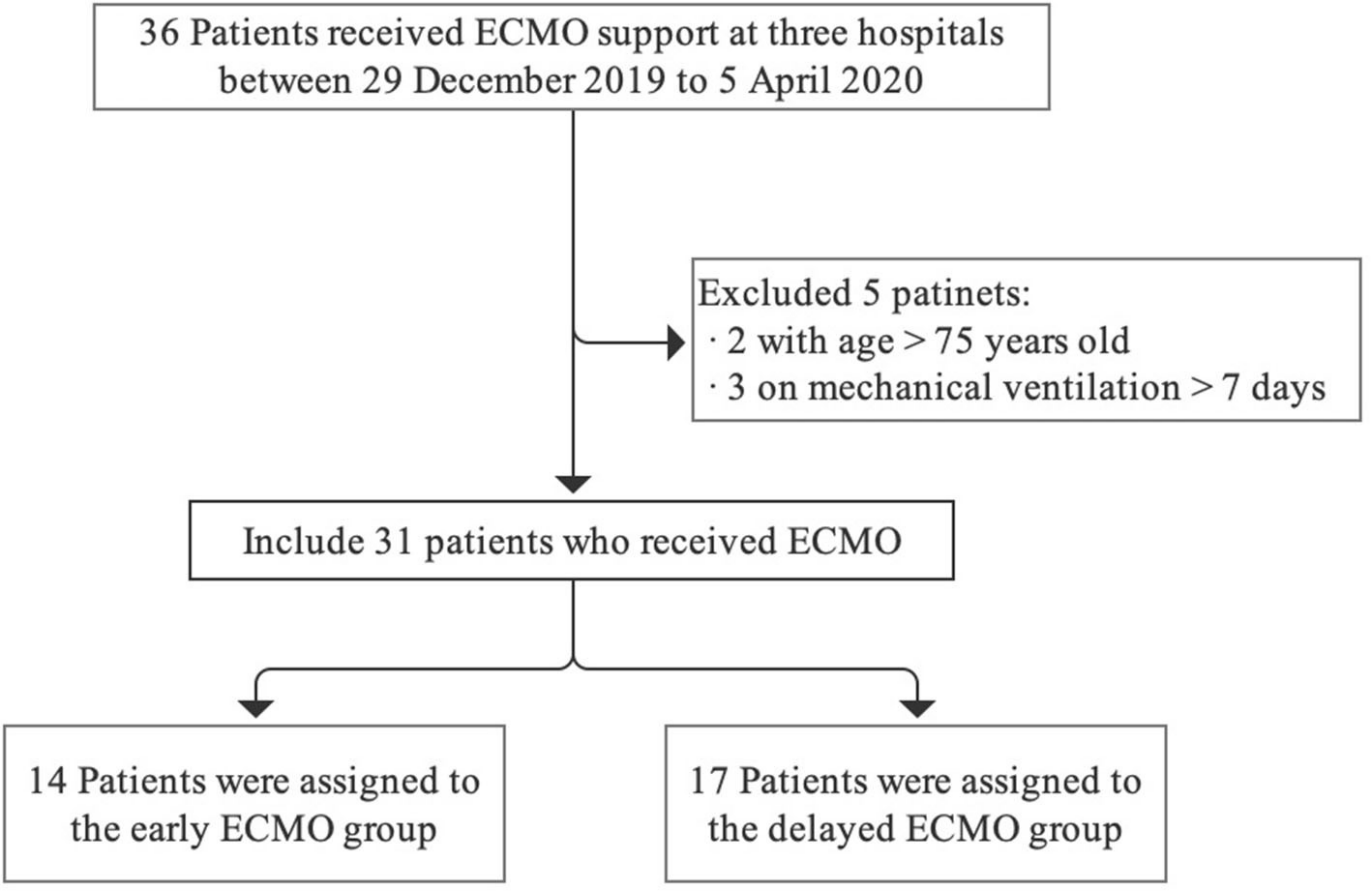
Flowchart of the study population.

Finally, 31 patients who received VV-ECMO support at the three hospitals were included in the analysis. Fourteen patients were assigned to the early ECMO group, and 17 patients were assigned to the delayed ECMO group. The flowchart of the study population is shown in Figure 1.

**Figure 2:**
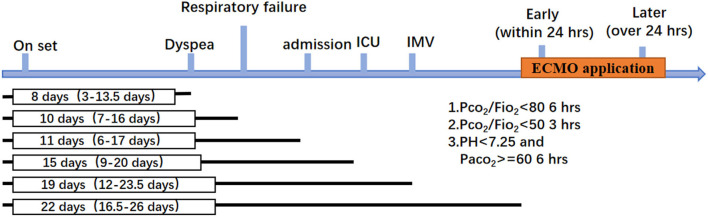
Clinical courses from illness onset in the included patients.

**Figure 3:**
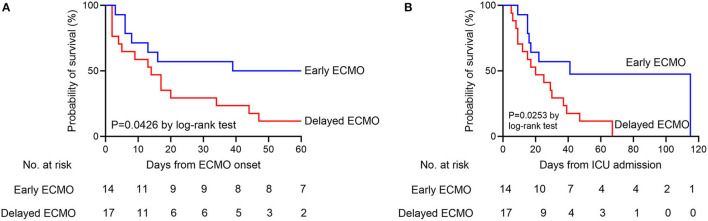
**(A)** Kaplan–Meier survival estimates of the early ECMO group and delayed ECMO group during the 60 days after ECMO onset. **(B)** Kaplan–Meier survival estimates of the early ECMO group and delayed ECMO group during the ICU stay. ECMO, extracorporeal membrane oxygenation; ICU, intensive care unit.

Among the 31 patients included in the analysis, the median age was 58 years (IQR, 46–64.5 years), and 19 (61%) were men. The recorded comorbidities included hypertension *n* = 7 (23%) and diabetes *n* = 5 (16%), and no difference between the two groups was observed. The Acute Physiology and Chronic Health Evaluation (APACHE II) scores of the patients were not significantly different between the groups both on ICU admission and before ECMO application. The Sequential Organ Failure Assessment (SOFA) score before ECMO application was not significantly different between the groups (*P* > 0.999). The median length from symptom onset to hospital admission was 11 days (IQR, 6–17 days), and the median time from symptom onset to ICU admission, to IMV and to ECMO were 15 days (IQR, 9–20 days), 19 days (IQR, 12–23.5 days), and 22 days (IQR, 16.5–26 days), respectively. The clinical course is shown in Figure 2. Patients in the early ECMO group had a significantly shorter duration of IMV before ECMO, duration of mechanical ventilation (MV) before ECMO, and duration of HFNC and MV before ECMO than the values in the delayed ECMO group (Table 1). The time between meeting the ECMO criteria and the initiation of ECMO was significantly longer in the delayed ECMO group (*P* < 0.001). Regarding the outcomes, delayed initiation of ECMO was associated with increased 60-day mortality after ECMO compared with the early ECMO group (88 vs. 50%, *P* = 0.044). The ICU mortality was also increased in the delayed ECMO group (94 vs. 57%, *P*=0.026). Kaplan–Meier survival estimates during the 60 days after ECMO onset and ICU stay showed statistical significance between the groups (*P* = 0.0426 and 0.0253, respectively) (Table 1, Figure 3). Fewer patients in the delayed ECMO group were successfully weaned from ECMO than in the early ECMO group (6 vs. 50%, *P* = 0.011). The median duration of ECMO was 14 days (IQR, 4.5–35 days). The total viral nucleic acid negative conversion ratio and viral shedding days were similar between the groups.

**Table 1:** Demographics and clinical course of patients treated with ECMO.

	**Total**	**Early ECMO**	**Delayed ECMO**	***P***
	**(*n* = 31)**	**(*n* = 14)**	**(*n* = 17)**	
**Demographics**				
Age, year	58 (46–64.5)	58.5 (42–65)	57 (47–65)	0.815
Male, *n* (%)	19 (61%)	12 (86%)	7 (41%)	0.011
Ever smoker, *n* (%)	4 (13%)	2 (14%)	2 (12%)	>0.999
**Comorbidities**, ***n*****(%)**				
Hypertension	7 (23%)	4 (29%)	3 (18%)	0.671
Diabetes	5 (16%)	2 (14%)	3 (18%)	>0.999
**On ICU admission**				
APACHE II score	12.4 ± 3.8	13.4 ± 4.6	11.6 ± 3.0	0.184
**Before ECMO**				
APACHE II score	13.0 ± 3.8	13.6 ± 4.1	12.6 ± 3.6	0.469
Lung injury score	4 (3, 4)	3.5 (3, 4)	4 (3, 4)	0.706
SOFA score	9.0 ± 3.1	9.0 ± 3.4	9.0 ± 3.1	>0.999
**Clinical course**				
Symptom onset to dyspnea, day	8 (3–13.5)	9.5 (4–13)	6 (2–14)	0.815
Symptom onset to respiratory failure, day	10 (7–16)	13 (6–17.5)	9 (7–15)	0.872
Symptom onset to hospital admission, day	11 (6–17)	10 (6–15)	15 (8–20)	0.142
Symptom onset to ICU admission, day	15 (9–20)	16 (7.5–21)	15 (12–21)	0.706
Symptom onset to NIV, day	17 (9–21.5)	23 (14.5–26)	22 (18–29)	0.275
Symptom onset to IMV, day	19 (12–23.5)	21 (14–25)	19 (12–22)	0.506
Symptom onset to ECMO, day	22 (16.5–26)	23 (14.5–26)	22(18–29)	0.706
**Respiratory support before ECMO**				
HFNC, *n* (%)	16 (52%)	8 (57%)	8 (47%)	0.576
NIV, *n* (%)	17 (55%)	5 (36%)	12 (71%)	0.052
Prone position, *n* (%)	19 (61%)	7 (50%)	12 (71%)	0.242
Duration of HFNC, day	2.5 (2–5)	3 (1–4)	2 (2–6)	0.932
Duration of NIV, day	3.5 (2–7)	3 (2–6)	4 (1.5–12)	0.643
Duration of IMV before ECMO, day	2 (1–4)	1 (0–2)	4 (2–6)	<0.001
Duration of HFNC and NIV before IMV, day	3 (2–8.5)	3 (2.5–6)	4 (2–11)	0.418
Duration of NIV + IMV before ECMO, day	4 (2–8.5)	2.5 (1–5.5)	7 (3–15)	0.006
Duration of NIV + HFNC + IMV before ECMO, day	7 (3–12.5)	3.5 (3–8)	9 (5–15)	0.024
**First indication of ECMO met during IMV**				
PaO_2_/FiO_2_ ratio <80 mmHg for 6 h	23 (74%)	9 (64%)	14 (82%)	0.412
PaO_2_/FiO_2_ ratio <50 mmHg for 3 h	8 (26%)	4 (29%)	4 (24%)	>0.999
pH <7.25 and PaCO_2_ ≥ 60 mmHg for 6 h	3 (10%)	1 (7%)	2 (12%)	>0.999
Time between meeting the ECMO criteria and the initiation of ECMO, day	2 (1–4)	0.5 (0–1)	3 (2–5)	<0.001
**Outcome**				
Duration of IMV, day	19 (7.5–40)	32.5 (8–43.5)	17 (6–38)	0.397
Duration of ECMO, day	14 (4.5–35)	20.5 (7.5–39.5)	13 (2–20)	0.114
Viral nucleic acid negative conversion ratio, *n* (%)	17 (55%)	9 (64%)	8 (47%)	0.337
Viral shedding, day	27 (19.5–30.5)	28 (23.5–39.5)	24 (18–29)	0.200
Weaning from ECMO, *n* (%)	8 (26%)	7 (50%)	1 (6%)	0.011
60-day mortality after ECMO, n (%)	22 (71%)	7 (50%)	15 (88%)	0.044
ICU mortality, *n* (%)	24 (77%)	8 (57%)	16 (94%)	0.026

*ECMO, extracorporeal membrane oxygenation; APACHE, Acute Physiology and Chronic Health Evaluation; SOFA, Sequential Organ Failure Assessment; ICU, intensive care unit; IMV, invasive mechanical ventilation; NIV, non-invasive ventilation; HFNC, high-flow nasal cannula oxygen therapy; PaO_2_, partial pressure of arterial oxygen; FiO_2_, fraction of inspired oxygen; PaCO_2_, partial pressure of arterial carbon dioxide.*

*The enumeration data indicators were described by frequency/percentage and were compared by chi-square test or Fisher's exact test. The continuous variables with normal distribution were described by mean ± SD, and variables with non-normal distribution were described by median and interquartile ranges (IQRs). Variables with a normal distribution were compared by Student's t-test, and variables with a non-normal distribution were compared by the Mann–Whitney test. Significance was set as P < 0.05.*

*Early initiation of ECMO was defined as initiation of ECMO within 24 h once met with one of the first indications of ECMO initiation.*

*The first indication of ECMO initiation during IMV was defined as the earliest timepoint at which patients met one of the three criteria.*

*(1) Ratio of partial pressure of arterial oxygen (PaO_2_) to the fraction of inspired oxygen (FiO_2_) of <50 mm Hg more than 3 h.*

*(2) PaO_2_/FiO_2_ of <80 mm Hg for more than 6 h; and.*

*(3) Arterial blood pH of <7.25 with a partial pressure of arterial carbon dioxide (PaCO_2_) of at least 60 mm Hg for more than 6 h*.

### Parameters and Physiological Indicators Pre- and During ECMO

Patients receiving ECMO treatment were followed for 6 h before ECMO application and 24 and 72 h after ECMO. Three patients died between 24 and 72 h after ECMO application; thus, their data are missing from the 72 h follow-up. The respiratory rate improved after ECMO application, dropping from 30 ± 14 to 25 ±8 beats/min (bpm) 24 h after ECMO and 22 ± 7 bpm 72 h after ECMO (Table 2). The mean arterial blood pressure (MAP) was rather steady at >75 mmHg. The initial tidal volume (Vt/predicted body weight) was 5.2 ± 1.0 ml/kg before ECMO onset and was reduced to 3.2 ± 0.9 ml/kg after ECMO onset, in compliance with the “lung protective ventilation strategy.” A relatively high positive end expiratory pressure (PEEP) was seen before and after ECMO application, although the differences were not observed between each time point. Patient oxygenation was significantly improved after the onset of ECMO. The PaO_2_ was 66 ± 19 mmHg before ECMO onset and 89 ± 31 and 82 ± 23 mmHg at each time point after ECMO onset, and both were significantly improved compared with 6 h pre-ECMO (*P* = 0.003). The PaO_2_/FiO_2_ ratio was 76 ± 29 before ECMO, 146 ± 70 at 24 h, and 163 ± 92 at 72 h (*P* < 0.001). Carbon dioxide (CO_2_) clearance was also significantly improved (6 h pre-ECMO 56 ± 20 mmHg vs. 24 h on ECMO 43 ± 12 mmHg vs. 72 h on ECMO 41 ± 6 mmHg, *P*=0.001).

**Table 2:** Parameter and physiological indicators pre-ECMO and on ECMO.

	**6 h pre-ECMO** **(*N* = 31)**	**24 h on ECMO ** **(*N* = 31)**	**72 h on ECMO** **(*N* = 28)**	***P***
T, °C	37.4 ± 0.9	36.9 ± 0.5^a^	36.8 ± 0.4^b^	0.003
RR, bpm	30 ± 14	25 ± 8	22 ± 7	0.037
HR, bpm	112 ± 27	109 ± 23	109 ± 21	0.668
MAP, mmHg	76 ± 15	77 ± 16	76 ± 15	0.878
Vt/predicted body weight, ml/kg	5.2 ± 1.0	3.2 ± 0.9^a^	3 ± 0.8^b^	<0.001
PEEP, cmH_2_O	13 ± 3	12 ± 3	12 ± 2	0.523
Compliance, ml/cmH_2_O	20 ± 3	NA	NA	NA
Lactate, mmol/L	2.2 ± 1.2	2.7 ± 3.0	2.6 ± 3.0	0.727
SaO_2_, %	87 ± 12	92 ± 11^a^	94 ± 5^b^	0.006
PaO_2_, mmHg	66 ± 19	89 ± 31^a^	82 ± 23^b^	0.003
PaO_2_/FiO_2_ ratio	76 ± 29	146 ± 70^a^	163 ± 92^b^	<0.001
PaCO_2_, mmHg	56 ± 20	43 ± 12^a^	41 ± 6^b^	0.001
pH	7.39 ± 0.11	7.40 ± 0.10	7.38 ± 0.15	0.767
ECMO blood flow, (L/min)	NA	4.58 ± 0.34	4.54 ± 0.33	0.640
ECMO sweep gas flow, (L/min)	NA	4.77 ± 0.51	4.72 ± 0.48	0.774

*ECMO, extracorporeal membrane oxygenation; T, temperature; SD, standard deviation; RR, respiratory rate; HR, heart rate; MAP, mean arterial pressure; Vt, tidal volume; PEEP, positive end-expiratory pressure; SaO2, arterial oxygen saturation; PaO2, partial pressure of arterial oxygen; FiO2, fraction of inspired oxygen; PaCO2, partial pressure of arterial carbon dioxide; NA, not applicable. Sequential parameters were compared by repeated measures ANOVA. ^a^P < 0.05 for 24 h on ECMO compared with 24 h pre-ECMO; ^b^P < 0.05 for 72 h on ECMO compared with 24 h pre-ECMO.*

*Missing data due to deceased patients at 72 h on ECMO were replaced with data at 24 h on ECMO in the repeated ANOVA*.

### Patient laboratory Findings and Organ Function Before and During ECMO Onset

Routine blood tests showed a trend of decreased total white blood cell count but were not significantly different between each time point. The lymphocyte count was 0.5 × 10^9^/L (0.3–0.7) before ECMO and 0.6 × 10^9^/L (0.5–1.1) 72 h after ECMO (Supplementary Table 1). Although blood transfusion was actively implemented in most of the patients, hemoglobin showed a sequential downward trend. A significant reduction was observed in the level of platelets at 72 h on ECMO (*P* < 0.001). A mildly elevated total bilirubin (TBIL) level was also observed. The creatinine level was slightly elevated after ECMO but was not significantly different between timepoints. Regarding laboratory findings on coagulopathy, a significantly prolonged activated partial thromboplastin time (APTT) was noticed after ECMO initiation due to the continuous infusion of heparin. APTT was continuously monitored and was fluttered from 51.8 ± 20.2 to 54.4 ± 16.0 s after ECMO. The decreased fibrinogen (FIB) was also observed in patients after ECMO initiation (2.8 ± 1.7 g/L at 24 h on ECMO; 3.0 ± 1.9 g/L at 72 h on ECMO). D-dimer was not significantly different among the timepoints (Supplementary Table 2).

### Treatment of Patients Using ECMO

In the total 31 patients, more than half of the patients received antiviral treatment [21 (68%)] (Table 3). A continuous renal replacement therapy (CRRT) was applied 27 times for the clearance of inflammatory mediators and renal function protection. Four (16%) patients received tracheotomy. Significant differences were not observed between the groups.

**Table 3:** Treatments of patients using ECMO.

	**Total** **(*n* = 31)**	**Early ECMO** **(*n* = 14)**	**Delayed ECMO** **(*n* = 17)**	***P***
Antiviral agents, *n* (%)	21 (68%)	9 (64%)	12 (71%)	>0.999
Oseltamivir	6 (19%)	2 (14%)	4 (24%)	0.664
Ganciclovir	4 (13%)	1 (7%)	3 (18%)	0.607
Lopinavir/ritonavir	17 (55%)	8 (57%)	9 (53%)	0.815
Arbidol	7 (23%)	4 (29%)	3 (18%)	0.671
Ribavirin	2 (7%)	1 (7%)	1 (6%)	>0.999
Remdesivir/placebo	1 (3%)	0 (0%)	1 (6%)	>0.999
CRRT, *n* (%)	27 (87%)	13 (93%)	14 (82%)	0.607
CRRT duration, day	8 (3–19.5)	8 (3.5–30)	8 (2–13.5)	0.705
Tracheotomy, *n* (%)	5 (16%)	2 (14%)	23(18%)	>0.999

*ECMO, extracorporeal membrane oxygenation; NIV, non-invasive ventilation; HFNC, high-flow nasal cannula oxygen therapy; CRRT, continuous renal replacement therapy*.

### Complications in Patients Using ECMO

In total, ventilation-associated pneumonia (VAP) occurred in 22 (71%) patients (Table 4). Catheter-related bloodstream infection (CRBSI) occurred in eight (26%) patients. Nine patients had hemorrhage onset during the clinical course, and one patient had bleeding at multiple sites. Barotrauma occurred in eight (26%) patients and was related to IMV. Hemolysis was seen in one patient related to ECMO. The ECMO mechanical complications were seen in six patients.

**Table 4:** Complications of patients treated with ECMO.

	**Total** **(*n* = 31)**	**Early ECMO** **(*n* = 14)**	**Delayed ECMO** **(*n* = 17)**	***P***
**Nosocomial infection**				
VAP, *n* (%)	22 (71%)	9 (64%)	13 (77%)	0.693
CRBSI, *n* (%)	8 (26%)	5 (36%)	3 (18%)	0.412
Hemorrhage, *n* (%)	9 (29%)	3 (21%)	6 (35%)	0.456
Gastrointestinal bleeding, *n* (%)	9 (29%)	3 (21%)	6 (35%)	0.456
Tracheotomy wound hemorrhage, *n* (%)	1 (3%)	0 (0%)	1 (6%)	> 0.999
Intracerebral hemorrhage, *n* (%)	1 (3%)	0 (0%)	1 (6%)	> 0.999
Barotrauma, *n* (%)	8 (26%)	2 (14%)	6 (35%)	0.240
Hemolysis, *n* (%)	1 (3%)	1 (7%)	0 (0%)	0.452
Thrombocytopenia, *n* (%)	15 (48%)	6(43%)	9 (53%)	0.576
**ECMO mechanical complications**				
Oxygenator failure, *n* (%)	2 (7%)	0 (0%)	2 (12%)	0.488
Oxygenator thrombosis, *n* (%)	4 (13%)	2 (14%)	2 (12%)	> 0.999
Sepsis shock, *n* (%)	14 (45%)	5 (36%)	9 (53%)	0.337

*ECMO, extracorporeal membrane oxygenation; VAP, ventilator-associated pneumonia; CRBSI, catheter-related bloodstream infection*.

### Characteristics of Eight Successfully Weaned ECMO Patients

Eight patients were successfully weaned off ECMO, and among them, one patient died 10 days after weaning because of severe infection due to invasive pulmonary aspergillosis (IPA). Seven (88%) of these patients were men, with a median age of 44.5 years (IQR, 41–63 years) (Supplementary Table 4). The pre-ECMO APACHE II score and SOFA score were 12.6 ± 4.4 and 7.9 ± 3.0, respectively. Median ECMO duration was 26 days (IQR, 17–38 days). All patients received IMV before ECMO. The PaO_2_/FiO_2_ ratio was 219 ± 93 mmHg, and the partial pressure of arterial carbon dioxide (PaCO_2_) was 41 ± 9 mm Hg before weaning (Supplementary Table 3).

## Discussion

This study descriptively discussed and elaborated on the efficacy and safety of ECMO in patients with COVID-19 with severe ARDS. We found that the outcome was related to the timing of ECMO initiation, and patients in the early initiation group had an increased ECMO successful weaning rate and decreased 60-day mortality after ECMO compared to the delayed ECMO patients.

A majority (67–85%) of critically ill patients admitted to ICUs with confirmed COVID-19 developed ARDS (5, 20). Although the Extracorporeal Life Support Organization (ELSO) issued guidance on the use of ECMO in patients with COVID-19 (21), ECMO should be considered according to the standard management algorithm for ARDS in supporting patients with viral lower respiratory tract infection. Many studies also recommend ECMO as a standard strategy in experienced ECMO centers for patients meeting EOLIA criteria (22). However, the worldwide experience with using ECMO to support patients with COVID-19 is limited. Therefore, we conducted this study to report on the efficacy and safety of ECMO in patients with severe ARDS related to COVID-19.

The application of ECMO could improve oxygenation, reduce carbon dioxide, and stabilize the vital signs of patients. However, the 60-day mortality after ECMO was 71% in this study, which was higher than the mortality of H1N1-induced ARDS following the ECMO treatment (8, 23) and even slightly higher than the mortality of severe MERS-CoV and H7N9 pneumonia patients treated by ECMO (9, 10). The main reason was the controversial results regarding the timing of ECMO initiation.

Although patients may meet the EOLIA criteria, which are widely used, ECMO support may not be initiated in time (7, 24). The COVID-19 pandemic has greatly strained the critical care resources of hospitals in Wuhan, especially if they were not adequately resourced or staffed (5). In the early stage of the outbreak, we focused on increasing the number of beds, ventilators, and ICU clinicians to address the problems. This study found that 55% of the patients were in the delayed ECMO group, and ECMO support was established at a median of 4 days after meeting the EOLIA criteria. They had higher 60-day mortality after ECMO and a lower successful weaning rate. Compared to the early group, they had a longer duration of IMV before ECMO. The duration of IMV before ECMO of more than 7 days was an important prognostic factor for death (25). Although the IMV duration of all patients pre-ECMO in our study was <7 days, the delayed group had a significantly longer duration, with a median of 4 days before ECMO. However, ECMO was started at 2 h (1–5 h) after IMV among the patients with H1N1, with a lower ICU mortality of 23% in Australia and New Zealand in 2009 (8). Therefore, we emphasize the importance of early implementation of ECMO in patients with severe ARDS related to COVID-19.

The past experience with the self-limited disease H1N1 or H7N9 virus-induced ARDS indicated that the median duration of ECMO support was 8–10 days (8, 9, 26). For all types of ARDS, the EOLIA study showed that the median duration was 15 days (7). However, the median duration was 26 days in successfully weaned COVID-19 patients, which was partially related to the prolonged viral shedding and lack of a clear antiviral therapy in SARS-CoV-2 (13). ELSO-issued guidance showed that ~21 days on ECMO could be considered futile, and this study indicated that extending the duration of ECMO should be considered for the patients infected with COVID-19 (21).

The hospital-level volume of ECMO cases was related to the outcomes of patients (27), and VV-ECMO for respiratory support in Wuhan was not at the leading level before this outbreak in China. Despite the support of medical staff from national high-level ECMO treatment centers, management problems were still one of the risk factors leading to high mortality. Nosocomial infections during ECMO were associated with longer duration (28, 29), and VAP was the most common nosocomial infection in this study. According to the autopsy results for COVID-19 (30), SARS-CoV-2 seldom infiltrates the lungs with lymphocytes, which is different from influenza virus pneumonia. SARS-CoV-2 invades the lungs, hearts, kidneys, and other organs, while immune organs, such as the spleen, show “desolation.” Immunological status should be considered when selecting candidates for ECMO. The incidence of nosocomial infection was above the normal level because of damage to the immune function of the patients and inadequate prevention due to the state of the COVID-19 outbreak. Therefore, intensification of airway management and reasonable management of catheters, such as deep vein catheters, are of vital importance.

The incidence of hemorrhage and oxygenator thrombus was high, which suggested that some problems existed in our anticoagulation management and organ supportive treatment of ECMO (31). Thus, coagulation tests, such as the activated clotting time (ACT) or APTT, should be regularly monitored to avoid large fluctuations, and invasive operations, such as tracheotomy, should be performed in the early stage of ECMO to prevent fatal factors, such as intratracheal massive hemorrhage.

In addition to the “lung protective ventilation strategy” (32), patients with severe COVID-19-related ARDS are often given higher PEEP (33) and placed in the prone position (34). The effects of lung recruitment and titrated PEEP are still unclear in patients with pulmonary endogenous ARDS caused by pneumonia (35). During ECMO support, the “lung rest strategy” was mainly used (36, 37) due to the high clinical workload and constrained environment. An individual patient data meta-analysis of observational studies in ventilated patients with ARDS receiving ECMO for refractory hypoxemia showed that the driving pressure during ECMO was the only ventilator setting that showed an independent association with in-hospital mortality (38). Unfortunately, some specific information from the ICU was missing, and many patients did not have driving pressure measurements. There were four pneumothorax patients during ECMO support. Perhaps due to the special respiratory mechanics of patients with COVID-19, the main findings were low respiratory system compliance and poor reactivity to recruitment maneuvers with high PEEP in a single-center observational study (39). Therefore, the optimal ventilation strategy is still unclear.

This study has several limitations. First, only 31 ECMO patients were included in this study. All ECMO patients cared for in the ICU of three hospitals who met the inclusion criteria were included. With the increased number of patients with COVID-19, we hope that these findings presented here will promote a larger cohort study or potentially some randomized controlled trials. Second, the data collected were only from the ICUs of three hospitals, which may not reflect the treatment of severe ECMO patients in the whole region of Wuhan, thereby resulting in the data selection bias. Third, this is a retrospective study, and the number of subjects was too small to perform a multiple regression analysis to identify the risk factors for unsuccessful weaning from ECMO. Therefore, further studies are still needed.

## Conclusion

Extracorporeal membrane oxygenation is effective at improving oxygenation and ventilation of patients with severe ARDS related to COVID-19, and early application successfully improves weaning and increases survival compared with the delayed initiation of ECMO. As the duration of ECMO in successfully weaned patients was prolonged, it was necessary to prevent and control complications, such as nosocomial infection and hemorrhage, to generate appropriate conditions for lung recovery.

## Data Availability Statement

The raw data supporting the conclusions of this article will be made available by the authors, without undue reservation.

## Ethics Statement

The study was approved by Jinyintan Hospital ethics board. Informed consent was waived since we collected and analyzed all data from the patients according to the policy for public health outbreak investigation of emerging infectious diseases issued by the National Health Commission of the People's Republic of China.

## Author Contributions

XL, MH, RZ, HQ, BD, and ZT: conception, design, and administrative support. XL, MH, RZ, LJ, MZ, LS, XZ, CP, WZ, HQ, BD, and ZT: provision of study materials or patients. XL, YW, HK, LJ, MZ, LS, XZ, CP, and WZ: collection and assembly of data. XL, MH, RZ, YW, and HK: data analysis and interpretation. All authors manuscript writing and final approval of manuscript.

## Conflict of Interest

The authors declare that the research was conducted in the absence of any commercial or financial relationships that could be construed as a potential conflict of interest.

## Publisher's Note

All claims expressed in this article are solely those of the authors and do not necessarily represent those of their affiliated organizations, or those of the publisher, the editors and the reviewers. Any product that may be evaluated in this article, or claim that may be made by its manufacturer, is not guaranteed or endorsed by the publisher.

## Abbreviations

ECMO: extracorporeal membrane oxygenation
COVID-19: coronavirus disease 2019
IMV: invasive mechanical ventilation
SARS-CoV: severe acute respiratory syndrome coronavirus
MERS-CoV: Middle East respiratory syndrome coronavirus
RNA: ribonucleic acid
ARDS: acute respiratory distress syndrome
H1N1: influenza A
H7N9: avian influenza A
VV-ECMO: veno-venous ECMO
WHO: World Health Organization
ICU: intensive care unit
HFNC: high-flow nasal cannula oxygen therapy
NIV: non-invasive ventilation
PaO2: partial pressure of arterial oxygen
FiO2: fraction of inspired oxygen
SD: standard deviation
IQR: interquartile range
ANOVA: analysis of variance
APACHE II: Acute Physiology and Chronic Health Evaluation
SOFA: Sequential Organ Failure Assessment
MV: mechanical ventilation
MAP: mean arterial blood pressure
Vt: tidal volume
PEEP: positive end expiratory pressure
CO2: carbon dioxide
TBIL: total bilirubin
APTT: activated partial thromboplastin time
FIB: fibrinogen
CRRT: continuous renal replacement therapy
VAP: ventilation associated pneumonia
CRBSI: catheter related blood stream infection
IPA: invasive pulmonary aspergillosis
PaCO2: partial pressure of arterial carbon dioxide
ELSO: Extracorporeal Life Support Organization
ACT: activated clotting time.
