# Supplementary material for: Delayed Initiation of ECMO Is Associated With Poor Outcomes in Patients With Severe COVID-19: A Multicenter Retrospective Cohort Study
Source: Front Med (Lausanne). 2021 Sep 16;8:716086. doi: 10.3389/fmed.2021.716086 (PMC8481658; doi:10.3389/fmed.2021.716086)
Supplement: Supplementary file 1 [file Table_1.DOCX]

**Additional file 1**

**The Berlin definition for diagnosis of ARDS**

1) Acute onset within 1 week of a clinical insult

2) Bilateral opacity on chest X-ray or computed tomography scan not fully explained by effusions, lobar/lung collapse, or nodules

3) Respiratory failure not fully explained by cardiac failure or fluid overload

4) A partial arterial oxygen pressure to fractional inspired oxygen concentration (PaO_2_/FiO_2_) ≤ 300

5) A minimum positive end-expiratory pressure (PEEP) of ≥ 5 cm H_2_O

**Reference**

ARDS Definition Task Force, Ranieri VM, Rubenfeld GD, et al. Acute respiratory distress syndrome: the Berlin Definition. JAMA.2012;307:2526-33.

**Table S1. Laboratory data pre and on ECMO**

|  | 6h pre-ECMO  (n=31) | 24h on ECMO  (n=31) | 72h on ECMO  (n=28) | *P* |
| --- | --- | --- | --- | --- |
| White blood cell, ×10^9^/L | 13.8±6.9 | 12.7±6.0 | 11.6±9.2 | 0.409 |
| Lymphocyte, ×10^9^/L | 0.5 (0.3-0.7) | 0.4 (0.3-0.8) | 0.6 (0.5-1.1) | 0.055 |
| Hemoglobin, g/L | 111.1±20.6 | 92.1±13.8*^a^* | 85.8±17.4*^b^* | ＜0.001 |
| Hematocrit, % | 34.3±5.9 | 28.4±4.1*^a^* | 26.4±5.5*^b^* | ＜0.001 |
| Platelet count, ×10^9^/L | 127.2±75.7 | 91.3±60.5*^a^* | 58.8±38.4*^b^* | ＜0.001 |
| ALT, IU/L | 34 (20.3-96.0) | 26 (16.3-84.8) | 32 (19.0-59.5) | 0.248 |
| AST, IU/L | 46 (30.8-67.0) | 45 (31.0-93.8) | 50 (34.5-102.5) | 0.347 |
| TBIL, μmol/L | 24.9±18.6 | 33.3±27.6 | 48.6±46.4 | 0.02 |
| DBIL, μmol/L | 11.1±10.8 | 15.6±18.5 | 26.8±34.7 | 0.037 |
| Urea, mmol/L | 11.0±5.4 | 13.2±5.4 | 12.8±7.8 | 0.283 |
| Creatinine, μmol/L | 111.0±73.4 | 131.8±77.2 | 131.9±87.9 | 0.368 |
| Creatine kinase, IU/L | 120 (69.3-289.3) | 295.5 (88.4-1186) | 249 (133.3-422.3) | 0.559 |
| Creatine kinase-MB, IU/L | 21 (14.9-33.2) | 23.1 (13.0-46.1) | 16.7 (13.2-27.5) | 0.223 |
| Albumin, g/dl | 32.8±5.7 | 31.7±5.3 | 34.4±5.1 | 0.088 |
| C-reactive protein, mg/L | 117.2±83.7 | 107.3±63.3 | 96.5±84.5 | 0.662 |
| APTT, s | 36.2±11.1 | 51.8±20.2*^a^* | 54.4±16*^b^* | ＜0.001 |
| D-dimer, μg/ml | 23.6±20.2 | 21.1±19.0 | 25.8±21.6 | 0.363 |
| Fibrinogen, g/L | 4.0±2.5 | 2.8±1.7 | 3.0±1.9 | 0.046 |
| PCT, ng/mL | 3.1±4.3 | 2.2±3.0 | 1.9±2.4 | 0.221 |

**Abbreviations:** ECMO, extracorporeal membrane oxygenation; ALT, alanine aminotransferase; AST, aspartate aminotransferase; TBIL, total bilirubin; DBIL, direct bilirubin; APTT, activated partial thromboplastin time; PCT, procalcitonin. Sequential parameters were compared by repeated measures analysis of variance (ANOVA). a. *P*＜0.05 24h on ECMO compared with 6h pre-ECMO; b. *P*＜0.05 72h on ECMO compared with 24h pre-ECMO

**Table S2. Organ dysfunction over time during ECMO among patients**

|  | D1 on ECMO  (n=31) | D3 on ECMO  (n=28) | D7 on ECMO  (n=21) | D14 on ECMO  (n=18) |
| --- | --- | --- | --- | --- |
| SOFA score | 9.9±3.2 | 10.2±3.3 | 9.3±3.4 | 8±3.4 |
| PaO_2_/FiO_2_ ratio | 148±72 | 153±96 | 158±57 | 222±122 |
| MAP, mmHg | 77±17 | 77±14 | 75±13 | 75±22 |
| High-dose vasoactive drugs, n (%) | 21 (68%) | 19 (68%) | 13 (62%) | 11 (61%) |
| Creatinine, μmol/L | 131.2±75.6 | 132.8±85.9 | 105.7±58.6 | 118.6±48.4 |
| Platelet count, ×10^9^/L | 90.4±58.3 | 57.4±38.1 | 78.1±45.9 | 94.4±61.6 |
| Bilirubin, μmol/L | 33.7±27.1 | 47.7±45.4 | 55.0±79.1 | 41.9±46.2 |
| GCS | 15 (15-15) | 15 (15-15) | 15 (15-15) | 15 (15-15) |

**Abbreviations:** ECMO, extracorporeal membrane oxygenation; SOFA, Sequential Organ Failure Assessment; PaO_2_, partial pressure of arterial oxygen; FiO_2_, fraction of inspired oxygen; MAP, mean arterial pressure; GCS, Glasgow coma scale.

**Table S3. Physiological indicator and laboratory test Pre-Successfully weaned**

|  | Pre-Successfully weaned  (n=8) |
| --- | --- |
| T, ℃ | 36.4±0.5 |
| RR, bpm | 22±3 |
| HR, bpm | 106±23 |
| MAP, mmHg | 84±17 |
| Vt / predicted body weight, ml/kg | 6.8±0.7 |
| PEEP, cmH_2_O | 7±3 |
| Compliance, mL/cmH_2_O | 31±3 |
| Lactate, mmol/L | 1.2±0.3 |
| SaO_2_, % | 98±3 |
| PaO_2_, mmHg | 101±50 |
| PaO_2_/FiO_2_ | 219±93 |
| PaCO_2_, mmHg | 41±9 |
| pH | 7.40±0.05 |
| White blood cell count, ×10^9^/L | 13.8±7.7 |
| Lymphocyte Count, ×10^9^/L | 1.3±0.6 |
| TBIL, μmol/L | 31.3±35.1 |
| Creatinine, μmol/L | 125.5±58.7 |
| Albumin, g/dl | 38.3±4.6 |
| C-reactive protein, mg/L | 89.1±16.3 |
| APTT, s | 50.2±14.1 |
| PCT, ng/mL | 3.1±1.9 |

**Abbreviations:** T, temperature; RR, respiratory rate; HR, heart rate; MAP, mean arterial pressure; Vt, tidal volume; PEEP, positive end-expiratory pressure; SaO_2_, arterial oxygen saturation; PaO_2_, partial pressure of arterial oxygen; FiO_2_, fraction of inspired oxygen; PaCO_2_, partial pressure of arterial carbon dioxide; TBIL, total bilirubin; APTT, activated partial thromboplastin time; PCT, procalcitonin.

**Table S4. Demographics and course of patient weaned from ECMO**

|  | **Successfully weaned**  **(n=8)** |
| --- | --- |
| **Demographics** |  |
| Age, y | 44.5 (41-63) |
| Male, n (%) | 7 (88%) |
| Comorbidities, n (%) |  |
| Hypertension | 1 (13%) |
| Diabetes | 2 (25%) |
| Ever smoker, n (%) | 0 (0%) |
| APACHE II score on ICU admission | 11.9±6.7 |
| APACHE II score pre-ECMO, | 12.6±4.4 |
| SOFA score pre-ECMO | 7.9±3.0 |
| Lung Injury Score | 3.0 (2.8-3.4) |
| **Clinical course** |  |
| Symptoms onset to hospital admission, day | 11 (4.5-18) |
| Symptoms onset to ICU admission, day | 17.5 (6-24) |
| Symptoms onset to NIV, day | 16.5 (6.5-23) |
| Symptoms onset to IMV, day | 19.5 (11-25) |
| Symptoms onset to ECMO, day | 22 (14.5-26) |
| Respiratory support before ECMO |  |
| Duration of IMV before ECMO, day | 2 (1-5) |
| Duration of NIV+HFNC+IMV before ECMO, day | 5 (3-9) |
| Earliest indication of ECMO initiation during IMV to ECMO onset, day | 0.5 (0-1) |
| IMV duration, day | 37 (32-46.5) |
| ECMO duration, day | 26 (17-38) |
| Viral nucleic acid negative conversion ratio, n (%) | 6 (75%) |
| Viral shedding, day | 33 (17.75-45.25) |

**Abbreviations:** ECMO, extracorporeal Membrane Oxygenation; SD, standard deviation; APACHE, Acute Physiology and Chronic Health Evaluation; SOFA, Sequential Organ Failure Assessment; ICU, intensive care unit; IMV, invasive mechanical ventilation; NIV, noninvasive ventilation.
